# The breast cancer paradox: A systematic review of the association between area-level deprivation and breast cancer screening uptake in Europe

**DOI:** 10.1016/j.canep.2019.03.008

**Published:** 2019-06

**Authors:** Dinah Smith, Katie Thomson, Clare Bambra, Adam Todd

**Affiliations:** a School of Pharmacy, Faculty of Medical Sciences, Newcastle University, King George VI Building, Newcastle 14 upon Tyne, NE1 7RU, UK; b Institute of Health and Society, Newcastle University, Richardson Road, Newcastle upon Tyne, NE2 4AX, UK

**Keywords:** Breast cancer, Screening, Health inequalities, Deprivation, Systematic review, Neighbourhood effects, Europe, Prevention, Public health

## Abstract

Breast cancer rates are lower amongst women from more socio-economically deprived areas. However, their mortality rates are higher. One explanation of this breast cancer paradox is that women from more deprived areas are less likely to attend breast cancer screening programmes. This systematic review is the first to examine this issue in Europe. A systematic review of Embase, Medline and PsychINFO (from 2008 to 2019) was undertaken (PROSPERO registration number: CRD42018083703). Observational studies were included if they were based in Europe, measured breast cancer screening uptake, compared at least two areas, included an area-level measure of socio-economic deprivation and were published in the English language. The Joanna Briggs Institute critical appraisal checklist was used to assess study quality and risk of bias. Thirteen studies from seven different European countries met our inclusion criteria and were included in the review. In ten of the thirteen studies, there was a significant negative association between screening uptake and area-level socio-economic deprivation – with women living in more socio-economically deprived neighbourhoods less likely to attend breast cancer screening. Although universal screening programmes were provided in most studies, there were still strong negative associations between screening uptake and area-level socio-economic deprivation. Future breast cancer screening strategies should acknowledge these challenges, and consider developing targeted interventions in more deprived areas to increase screening participation.

## Highlights

• Many countries in Europe offer breast cancer screening programmes.
• Our work identified 13 studies from 7 different European countries.
• Women from more deprived areas where less likely to attend breast cancer screening.
• Future screening strategies should consider developing strategies to account for this.

## 1 Introduction

Breast cancer is the most common type of cancer in women, accounting for 28% of total cancer cases across Europe [1]. In 2014, 92,500 women died from breast cancer in the EU-28 [2]. Through better diagnostic techniques and advancement in surgical and pharmacological treatment, mortality rates from breast cancer are decreasing [1]. However, there are substantial socio-economic inequalities in breast cancer prevalence and mortality: women in more socio-economically deprived areas have lower breast cancer incidence, but higher mortality rates [3]. For example, in England, incidence rates for breast cancer are 14% lower for women living in the most deprived areas, compared to the least deprived, while people living in the most deprived areas have a significantly higher mortality rate – with an estimated 350 yearly excess deaths [4]. This breast cancer paradox may be attributable to later diagnosis [5], suboptimal cancer care [6], co-morbidities that may limit treatment options or increase the possibility of developing treatment complications [7], and – most notably in terms of prevention – lower rates of breast cancer screening uptake.

In Europe, most countries have implemented or are developing mammography-based universal breast cancer screening programmes. It has been widely acknowledged that breast cancer screening is not without disadvantages, including breast cancer over diagnosis, which can lead to unnecessary surgical and pharmacological intervention. In view of this, many experts believe that the risks of breast cancer screening can outweigh the benefits, and women should always be fully informed about the risks and benefits of screening. Despite these limitations, the literature has shown that breast cancer screening can positively impact on survival [8]. However, even with universal screening programmes available in many European countries, there are still distinct area-based inequalities in breast cancer screening uptake related to socio-economic deprivation [[9], [10], [11], [12], [13]].

A systematic review by Pruitt et al. [14] examined the association between socio-economic deprivation and breast, cervical and colorectal cancer screening. The work included 13 studies for breast cancer screening – all of which were conducted in North America, and showed some positive associations between socio-economic status (SES) and screening uptake [14], indicating that as SES increased (i.e. increasing income and education, decreasing unemployment and poverty), the odds of attending cancer screening increased. However, no European studies were included in this systematic review and given the substantial differences in healthcare systems and screening coverage between the USA and Europe, it is important to examine if there is also an association in universal European health systems. Our systematic review, therefore, aimed to examine the association between area-level socio-economic deprivation and breast cancer screening uptake in Europe.

## 2 Materials and methods

### 2.1 Study design and inclusion criteria

The systematic review is registered with PROSPERO (CRD42018083703) and is reported according to the Preferred Reporting Items for Systematic Reviews and Meta-Analyses (PRISMA) guidelines [15] (Appendix A). We included observational studies (cross-sectional; prospective and retrospective cohorts, time series, repeat cross-sectional) of adult women (>18 years) in high-income European countries (defined as European countries in the Organisation of Economic Cooperation and Development). To be included in the review, studies had to compare at least two areas and have some area-level measure of socio-economic deprivation. Area-level socio-economic deprivation can be measured differently, but essentially involves ranking areas on the basis of relative local scores for factors such as income, employment and housing quality. Common measures include indices of multiple deprivation, percentage of poverty, or percentage unemployed. The primary outcome of interest was breast cancer screening participation. This included attending mammography, clinical breast examination (CBE), and ultrasound, but excluded self-breast examination. The screening could have been as either part of a screening programme, through referral by a healthcare professional, through health insurance or by any other certified method. In keeping with the previous systematic review by Pruitt et al. [14], studies had to be published in English and in peer-reviewed journals (to enhance quality); any studies only published as abstracts were excluded.

### 2.2 Search strategy

Our search terms are detailed in Table 1. The search strategy was adapted from Cairns et al. [16] who investigated the association of area-level socio-economic deprivation and suicidal behaviour, with additional breast cancer and screening terms. A pilot search was undertaken to identify three indicator papers [[17], [18], [19]]. Three electronic databases were searched between 1 st January 2008 and 28^th^ January 2019 (host sites given in parentheses): Medline (via OVID), Embase (via OVID) and PsycINFO (via OVID). The search start date of 2008 was chosen because our review updates the systematic review by Pruitt et al. [14]. Citation follow up of all included articles was also conducted.

**Table 1:** Search terms.

[(breast cancer AND mammo*) OR (breast cancer AND screen*) OR (mammo*) AND (socioeconomic OR SES OR education* OR employment OR income OR occupation* OR poverty OR class OR depriv* OR disadvantage* OR social class OR social factors OR economic OR unemployment) AND (area* OR geo* OR place OR neighbourhood OR neighbhorhood OR region* OR county OR ward OR city OR district OR county OR census tract OR metropolitan OR zip code)]

### 2.3 Data extraction and quality appraisal

Screening, data extraction and quality appraisal were conducted by two reviewers (DS and KT). Study inclusion agreement between the reviewers was 95% with a kappa score of fair (κ = 0.48) [20], as the first reviewer was more inclusive (all full texts were identified by both reviewers). The methods and main study findings of each study were extracted using a bespoke data extraction form. Data extraction and quality appraisal were conducted by DS and checked in full by KT; any discrepancies were resolved through discussion between the reviewers and then if agreement could not be reached by consensus through consulting the project lead (AT). Quality appraisal was conducted using the checklist for analytical cross sectional studies produced by the Joanna Briggs Institute (JBI) [21]. The checklist includes questions relating to sampling, inclusion criteria, confounding, types of outcomes and statistical analyses (Appendix B). Arbitrary labels of low (1–4), moderate (5 and 6) and high (7 and 8) were assigned to each primary study for ease of reporting.

### 2.4 Analysis and synthesis

A narrative synthesis thematically describing studies by country was undertaken. Studies used heterogeneous measures so meta-analysis was not possible. We report on the overall association between area-level deprivation and breast cancer screening uptake in terms of (1) the *gap* (between the best and the worst off areas); and (2) the *gradient* (across the whole spectrum of deprivation) [22].

## 3 Results

**Fig. 1:**
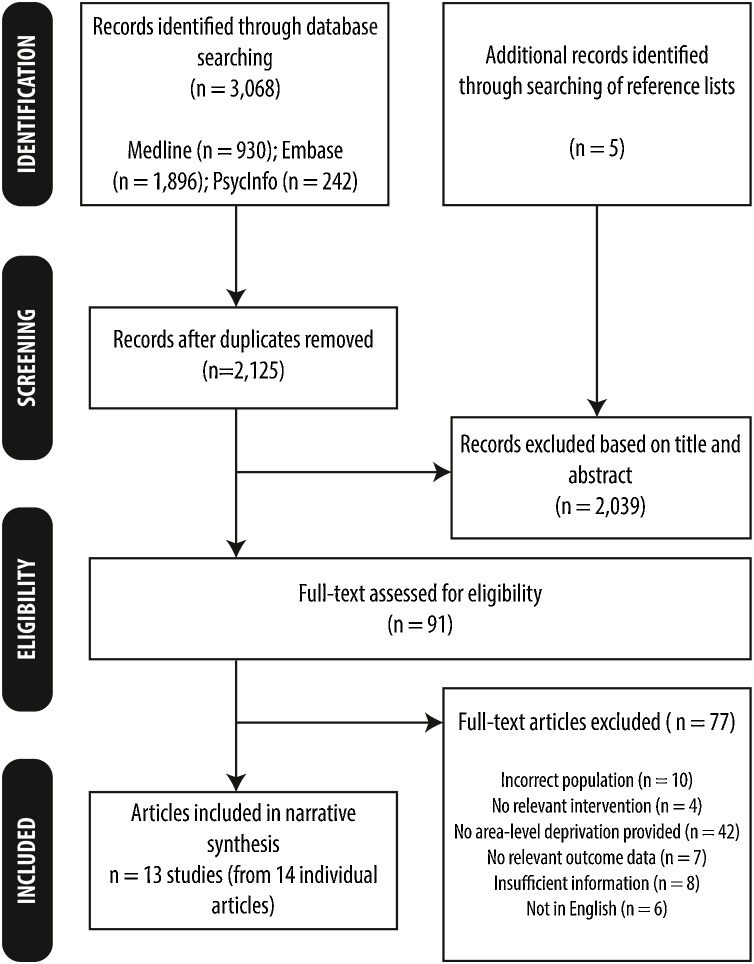
Prisma flow chart showing study selection.

A total of 2125 unique citations were retrieved from the searches. Reasons for exclusion at the full paper stage (n = 77) are detailed in Fig. 1 (with further details in Appendix C). In total, 13 unique primary studies (representing 14 articles) from seven countries were included in the review: England (n = 4, from 5 articles) [[23], [24], [25], [26], [27]], France (n = 3) [19,28,29], Germany (n = 1) [30], Italy (n = 1) [31], Sweden (n = 1) [18], the Netherlands (n = 1) [17] and Turkey (n = 2) [32,33]. All countries ran screening programmes, although it is acknowledged that, in Turkey, breast cancer screening is still performed primarily on an opportunistic basis [32,33]. The included studies examined screening uptake over different time intervals – the shortest of which was in Sweden [18] (1.5–2 years), while the longest screening interval was in England (3 years) [[23], [24], [25], [26], [27]]. The majority of the studies used small neighbourhood areas containing approximately 1500 residents called Lower Super Output Areas [25,27]; IRIS - French ‘aggregated units for statistical information’ [19,29]; or SAMS - Small Areas for Market Statistics [18]), although some used larger districts containing several hundred thousand residents (e.g. using English Primary Care Trust geographies [23,26]). Study size varied greatly: the largest comprised 4,805,390 women in all departments in mainland France [28], whilst the smallest was a single French department in the Normandy region of north-western France [29], involving 4940 women. Twelve of the thirteen studies examined mammography attendance [[17], [18], [19],[23], [24], [25], [26], [27], [28], [29], [30],^32^,^33^], whilst the other reported mammography and clinical breast examination attendance [34]. A variety of methods were employed in the primary studies to measure deprivation: most of the papers used composite measures of deprivation; for example the Index of Multiple Deprivation [[23], [24], [25], [26], [27]], the French European Deprivation Index [19,28], the Townsend Index [29,35], or other composite indices [17,18,34]; the other studies used single indicators [30,32,33]. The methodological quality of nearly half (6/13) of the included studies was high – with scores ranging from 2/8 (low) to 8/8 (high) (Appendix D).

### 3.1 Overall findings

**Fig. 2:**
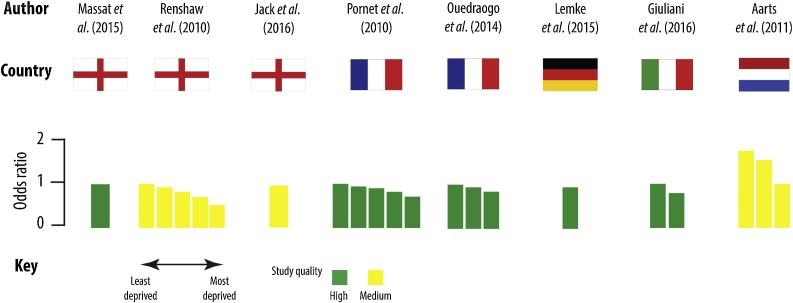
The association between area level socio-economic deprivation and breast cancer screening uptake for studies reporting odds ratios.

Regardless of indicator of area-level socio-economic deprivation, 11/13 studies (of which 10 were statistically significant) demonstrated a negative association between area-level deprivation and screening – with women living in more socio-economically deprived neighbourhoods less likely to attend breast cancer screening. Eight studies also examined the gradient (comparing more than two areas), and six demonstrated a socio-spatial gradient (three of which were statistically significant). The results for each study are narratively synthesised by country. Table 2 gives an overview of study characteristics and main findings, while Fig. 2 summarises the results of the studies that included odds ratios for breast cancer screening uptake.

**Table 2:** Summary table of included studies.

Study and source of funding		Design	Year(s)	Scale	Screening interval	Outcomes(s)	Measure of deprivation	Results (Confidence intervals given in square brackets)	Methodological quality	Deprivation effect	
										Gap (+/-)	Gradient (✓/ x)
**England**											
	Massat et al. [26] **Funding**: UK Department of Health	Cross-sectional	April 2011 – March 2012	District **Unit of analysis**: Primary Care Trusts	3 years	% of eligible women who had adequate mammography in the last 3 years	Percentage deprivation using the Index of Multiple Deprivation (IMD) 2010	*Model: Population and general practice-level variables* OR 0.991 [0.985, 0.997]	8/8 (high)	✓ – *	NR
	Renshaw et al. [27] **Funding**: Not listed	Cross-sectional	April 2004 – March 2007	Neighbourhood **Unit of analysis**: LSOA	3 years	Attendance at breast screening	Income quintile of the IMD 2004	% Sceening attendance given in brackets 1. (least deprived): OR 1.00 (69.2%) 2. OR 0.92 [0.91, 0.94] (67.5%) 3. OR 0.82 [0.81, 0.84] (64.9%) 4. OR 0.70 [0.69, 0.71] (61.1%) 5. OR 0.52 [0.51, 0.53] (53.8%)	6/8 (medium)	✓ – *	✓ – *
	Jack et al. [24,25] **Funding**: The London Quality Assurance Centre and the Thames Cancer Registry	Cross-sectional	March 2006 – December 2009	Neighbourhood **Unit of analysis**: LSOA	3 years	Women’s earliest invitation to screening was examined (split between first call invitation [for those women aged 50-52] and women aged 50-69 who had a routine recall invitation)	Income domain of the IMD 2007	2014 article: First call (% attended): 1 (most affluent): 66% 2 67% 3 63% 4 60% 5 56% Routine recall (% attended): 1 (most affluent): 79% 2 79% 3 77% 4 74% 5 70% 2016 article: OR 0.95 p<0.001	6/8 (medium)	[25]: First call: ✓ – NR Routine recall: ✓ – NR [24]: ✓ – *	[25]: First call: x Routine recall: ✓ NR [24]: **x**
	Douglas et al. [23] **Funding**: UK Department of Health	Cross-sectional	2007-2012	District **Unit of analysis**: Primary Care Trusts	3 years	% of eligible women who had adequate mammography in the last 3 years	IMD (2010) aggregated to PCT level	2007-2008 (average percentage coverage in brackets): 1 (least deprived): RR 1.00 (79%) 2. RR 0.99 [0.98, 1.00] (77%) 3. RR 0.94 [0.93, 0.95] (73%) 4. RR 0.94 [0.93, 0.95] (74%) 5. RR 0.85 [0.84, 0.86] (66%) 2012-13: 1 (least deprived): RR 1.00 (78%) 2. RR 1.00 [0.99, 1.01] (78%) 3. RR 0.96 [0.95, 0.97] (75%) 4. RR 0.95 [0.94, 0.96] (74%) 5. RR 0.89 [0.88, 0.90] (70%)	6/8 (medium)	2007-8: ✓ – * 2012-13: ✓ – *	2007-8: **x** 2012-13: **x**
**France**											
	Pornet et al. [29] **Funding**: Not reported	Cross-sectional	2004 – 2006	Neighbourhood **Unit of analysis**: IRIS	2 years	Screening mammography within the duration of the study	The Townsend Index	*Model: 2* 1 (least deprived): OR 1.00 (60.29%) 2. OR 0.94 [0.74, 1.20] (59.18%) 3. OR 0.90 [0.70, 1.14] (58.10%) 4. OR 0.82 [0.66, 1.02] (56.04%) 5. OR 0.71 [0.59, 0.86] (52.11%)	8/8 (high)	✓ – *	✓ NS
	Ouedraogo et al. [19] **Funding**: ‘La Ligue Contre le Cancer’ and ‘la ‘Fondation de France’	Cross-sectional	2010 – 2011	Neighbourhood **Unit of analysis**: IRIS	Not reported	Attended invitation to mammography screening between 2010 and 2011	The French European Deprivation Index	*Model*: *Multilevel logistic regression analyses* % Screening given in brackets 1 (least deprived): OR 1.00 (43.9%) 2. OR 0.94 [0.87, 1.02] (30.6%) 3. OR 0.84 [0.78, 0.92] (25.4%)	8/8 (high)	✓ – *	✓ NS
	Deborde et al. [28] **Funding**: Not reported	Cross-sectional	2013-2014	District **Unit of analysis**: Municipality	2 years	Age-standardised participation rate	The French Deprivation Index	% Attending screening: 1 (least deprived): 44.9% 2 54.2% 3 53.8% 4 54.8% 5 52.1%	6/8 (medium)	✓ + NR	**x**
**Germany**											
	Lemke et al. [30] **Funding**: Not reported	Cross-sectional	2007 - 2008, 2009 - 2010, 2011-2012	Neighbourhood **Unit of analysis**: Not specified	2 years	Participation in screening for each two year period	Unemployment rate (foreigner %)	*Model: Multivariable analysis* Unemployment rate (foreigner) OR 0.94 [0.90, 0.98]	7/8 (high)	✓ – *	NR
**Italy**											
	Giuliani et al. [34] **Funding**: San Paolo Foundation	Cross-sectional	1990 – 2000 (the time period patients were diagnosed with breast cancer), followed up until 2010	Neighbourhood **Unit of analysis**: Level of census section	Screening for breast cancer survivors	Yearly mammogram and CBE	Deprivation index	Affluent (reference) class: OR 1.00 Deprived class: OR 0.81 [0.65, 1.00]	8/8 (high)	✓ – NS	NR
**The Netherlands**											
	Aarts et al. [17] **Funding**: Bevolkings Onderzoek Borstkanker Zuid	Cross-sectional	1998 – 2005	Neighbourhood **Unit of analysis**: Postcode	2 years	Screening mammography attendance	Indicator of SES (based on house value and income)	Low SES: OR 1.00 (79%) Intermediate: OR 1.54 [1.5, 1.6] (85%) High: OR 1.75 [1.7, 1.8] (87%)	6/8 (medium)	✓ – *	✓ *
**Sweden**											
	Lagerlund et al. [18] **Funding**: Not reported	Cross-sectional	2005 – 2009	Neighbourhood **Unit of analysis**: SAMs	1.5-2 years	Non-attendance to most recent mammography screening during 2005 to 2009	Neighbourhood sociodemographic index	*Model: 3* Lowest % neighbourhood sociodemographic index (decile group 1: least deprived): OR 1.00 Decile group 5: OR 1.26 [1.07, 1.51] Highest % neighbourhood sociodemographic index (decile group 10): OR 1.92 [1.63, 2.34]	8/8 (high)	✓ – *	✓ *
**Turkey**											
	Dundar et al. [32] **Funding**: None	Cross-sectional	2008 – 2009	Regions **Unit of analysis**: Regions of Mansia	2 years	Attended screening between 2008 – 2009	Education, perceived family income	Attendees % Urban (70.5%), slum (81.6%)	2/8 (low)	✓ + *	NR
	Ozmen et al. [33] **Funding**: Not listed	Cross-sectional	Dates not stated	City **Unit of analysis**: City (Mus) and county (Bahcesehir) in Istanbul	Opportunistic screening	Screening mammography in the last two years	Literacy, graduation, working status, monthly income	Attendees % Mus – more deprived (35%) Bahcesehir (49%)	2/8 (low)	✓ – *	NR

OR indicates odds ratio.

RR indicates relative risk.

- indicates a negative association between area-level deprivation and breast cancer screening (i.e. the higher area-level deprivation, the lower the uptake of breast cancer screening).

- indicates a positive association between area-level deprivation and breast cancer screening (i.e. the higher area-level deprivation, the higher the uptake of breast cancer screening).

NR indicates data not reported in study.

*Significance at p < 0.05.

NS indicates gap/gradient not significant.

### 3.2 Country specific findings

#### 3.2.1 England (n = 4)

Four studies (from five articles) examined the association between area-level deprivation and uptake of the English Breast Screening Programme (one high methodological quality, three medium). All four studies [[23], [24], [25], [26], [27]] found a negative association between screening uptake and area-level deprivation. The papers by Jack et al. [24,25] observed both a gap and a gradient for the proportion of women who attended a first call for screening (significance not reported). The work by Massat et al. [26] found a statistically significant deprivation gap in uptake. Renshaw et al. [27] showed a gradient, whereby screening attendance was highest amongst the most affluent and fell significantly as deprivation levels increased. Douglas et al. [23] observed screening rates over a five year period and demonstrated a gap in all years and a gradient in all but one of the years. These findings suggest that there is a reduction in area-based screening inequalities from the years 2007–2008 to 2012-2013.

#### 3.2.2 France (n = 3)

Two of the three French studies (considered to have high methodological quality), found a negative association between deprivation and screening uptake. Ouedraogo et al. [19] and Pornet et al. [29] found a significant gap where women living in the most deprived neighbourhoods were significantly less likely than those living in the most affluent to participate in breast cancer screening. They also noted a gradient, but these were not statistically significant in either study. The study by Deborde et al. [28], using a larger unit of analysis (municipalities), found an inverted U-curve relationship between deprivation and screening participation, with highest screening found in the intermediate quintiles.

#### 3.2.3 Germany (n = 1)

The one high-quality study by Lemke et al. [30] found that in areas with a higher employment rate, women were more likely to attend breast cancer screening. However, this association was only statistically significant for foreigner unemployment rate, and not for other types of unemployment.

#### 3.2.4 Italy (n = 1)

A high-quality study by Giuliani et al. [34] only included participants who had been diagnosed with either in situ or invasive breast cancer and received surgical intervention. The results showed that those in the deprived group were less likely to attend yearly mammography screening and/or clinical breast examination compared to those in the reference group (but this gap was not statistically significant).

#### 3.2.5 Netherlands (n = 1)

Aarts et al. [17] explored breast cancer screening uptake in southern Netherlands by tertiles of deprivation. This medium-quality study found a statistically significant gap and gradient in breast cancer screening uptake.

#### 3.2.6 Sweden (n = 1)

Lagerlund et al. [18] explored non-attendance at mammography screening and neighbourhood sociodemographic characteristics in southwest Sweden. Both a gap and gradient effect was observed (significance not given) in this high-quality study: decreasing deprivation was associated with an increase in the proportion of women who attend screening appointments.

#### 3.2.7 Turkey (n = 2)

Two studies conducted in Turkey were identified, both of which were rated as low in quality. Dundar et al. [32] found that women in a more affluent, urban district were significantly less likely to attend the breast cancer screening programmes compared with those in the deprived district. Another study, by Ozmen et al. [33], compared the mammography screening behaviours of women in two socio-economically contrasting cities – with those in the more affluent city significantly more likely to have had a mammogram within the last two years.

## 4 Discussion

This review has found consistent, medium-high quality evidence of a negative association between area-level socio-economic deprivation and breast cancer screening uptake in Europe (i.e. as area level socio-economic deprivation increases, breast cancer screening uptake decreases). This work updates a previous systematic review exploring the association between breast cancer, colorectal cancer, and cervical cancer screening and area-level deprivation [14]. In this systematic review, which only included studies from outside Europe, thirteen studies focused on breast cancer screening; eight of which showed significant positive associations between SES and screening uptake. Our work, which focused on studies in Europe, supports the findings of the previous review by Pruitt et al. and provides evidence that even in the more universal health systems of Europe, women living in the least deprived areas are more likely to attend breast cancer screening than women living in the most deprived areas. Our work also builds on previous findings that as individual level SES increases, the uptake of breast cancer screening also increases [36,37], although the relationship between individual and area-level factors are thought to be complex with one not being a simple proxy for the other in terms of breast cancer risk [38]. Qualitative research provides contextual information to understand the reasons for low rates of screening in deprived areas. Evidence suggests that women living in areas of high deprivation had limited knowledge about mammography screening programmes or had misconceptions regarding cancer and mammography [12,39]. Embarrassment, fear and inconvenience have also been cited as possible reasons for low screening rates in these communities [40]. Furthermore, passive and practical barriers have been highlighted by an Australian Government Report [41] suggesting women in deprived communities find it difficult to attend mammography, because of transport (cost, availability) or family commitments.

It is possible that the reduced screening uptake in deprived areas contributes to the ‘breast cancer paradox’, whereby rates of breast cancer survival are reduced in deprived communities, despite there being lower disease incidence. Although there is debate about the role of breast cancer screening, screening does allow for early disease detection, which can result in earlier treatment initiation and better outcomes for patients. By not attending routine breast cancer screening, it is possible that the disease would be discovered at a more advanced stage, potentially resulting in poorer outcomes for patients. Although we acknowledge that reduced screening uptake in deprived communities is not solely responsible for the higher breast cancer mortality rate. With respect to disease incidence, high SES enhances certain behaviours that reduce breast cancer incidence, including reduced levels of smoking, lower alcohol consumption and increased physical activity. There are, however, other factors that increase breast cancer incidence in women of high SES, such as increased nulliparity, having children at an older age, and the use of the combined oral contraceptive pill [[42], [43], [44], [45]]. It is thought that these latter factors contribute to the increased incidence of breast cancer in women of higher SES.

Our findings are in line with the *fundamental cause theory* [46], whereby people with greater access to resources, including knowledge, money, power, prestige, and beneficial social connections are better able to take advantage of effective innovations in disease prevention and treatment. The theory predicts that, in people of higher SES, whenever technology and medical knowledge are available to manage a particular disease, the mortality rates of that disease will be lower. It is imperative then, that national breast cancer screening strategies across Europe and elsewhere, recognise the negative association of breast cancer screening uptake with area-level deprivation, and, potentially, how this could affect breast cancer mortality. Increasing knowledge, by targeting women of low socio-economic status and informing them of the benefits (and risks) of breast cancer screening and also providing practical steps to ensure mammography screening is fully accessible in deprived areas (e.g. by the use of mobile clinics) are thus potential routes to increase uptake. Small scale studies have demonstrated it is possible to achieve this; for example, Chambers et al. showed that a brief telephone support intervention doubled breast cancer screening attendance in women from deprived areas in Scotland who had missed their initial screening appointment [47]. Another study, undertaken by Kerrison et al. [48], showed that a text message reminder increased the uptake of routine breast cancer screening appointments for people living in the London Borough of Hillingdon. The findings of also suggested that text message reminders are particularly effective for increasing screening attendance among patients from deprived areas. Moving forward, it is important for these interventions to be tested at a national level and, if successful, be implemented into breast cancer screening policy.

This review is subject to some limitations. Firstly, we only included studies from European countries – we did not include studies from other high-income countries, such as the USA and Canada, nor did we analyse our findings according to healthcare system type [49,50]. Our findings, therefore, may not be generalisable to all types of healthcare systems. Secondly, this review is subject to the usual limitations of observational research whereby we cannot claim that there is a causal relationship between area-level disadvantage and breast cancer screening uptake. It is not clear how this association impacts on overall breast cancer survival, if at all – this limitation is even more pertinent in view of the debate around the risks and benefits of breast cancer screening [51]. Thirdly, other limitations due to practical resources include not being able to search for grey literature and unpublished studies. The studies included were also limited to the English language. Finally, as an evidence base we have only found a limited number of studies, and therefore it is possible that there is publication bias (that negative results are less likely to be published) and selective reporting within studies which may impact the assessment of risk of bias.

## 5 Conclusion

The findings from this review provide evidence that there is lower uptake of breast cancer screening in areas experiencing higher levels of socio-economic deprivation in Europe. Strategies to improve breast cancer screening attendance should be aimed at more deprived areas, to increase individual participation and decrease inequalities in the general population. Further research should explore how the reduced breast cancer screening uptake in areas of high socio-economic deprivation impacts on breast cancer survival and the breast cancer paradox.

## Funding

The project is supported by Fuse (UKCRC Centre for Translational Research in Public Health) (MRC grant ref. no. MR/K02325X/1). CB is a Fuse Senior Investigator, AT and KT are Associates and KT has been part-funded as a Research Associate. Funding for Fuse comes from the British Heart Foundation, Cancer Research UK, Economic and Social Research Council, Medical Research Council, and the National Institute for Health Research, under the auspices of the UK Clinical Research Collaboration, and is gratefully acknowledged. The funders had no role in study design, data collection and analysis, decision to publish, or preparation of the manuscript.

## Declarations of interest

None.

## Authorship contribution

DS, AT and CB contributed substantially to the conception and design of the project. DS and KT were responsible for the acquisition of data and the analysis. All authors contributed to the interpretation of data. All authors contributed to drafting the article and revising it critically for important intellectual content. All authors have provided the final approval of the version to be published.

## Availability of data and materials

All data generated or analysed during this study are included in this published article (and its additional files). The extraction forms for all the individual primary studies are available from the corresponding author on reasonable request.

## Ethics approval and consent to participate

Not applicable. Only published primary studies were included in this review.

## References

[1] World Health Organization (2018). Breast Cancer.

[2] Eurostat (2017). Cancer Statistics - Specific Cancers.

[3] Levi F., Lucchini F., Negri E., Vecchia C.L. (2004). Trends in mortality from major cancers in the European Union, including acceding countries, in 2004. Cancer.

[4] Cancer Research UK and National Cancer Intelligence Network (2014). National Cancer Intelligence Network: Cancer by Deprivation in England (Incidence, 1996-2010; Mortality, 1997-2011).

[5] MacKinnon J.A., Duncan R.C., Huang Y.J., Lee D.J., Fleming L.E., Voti L., Rudolph M., Wilkinson J.D. (2007). Detecting an association between socioeconomic status and late stage breast cancer using spatial analysis and area-based measures. Cancer Epidemiol. Biomarkers Prev..

[6] Bickell N.A., LePar F., Wang J.J., Leventhal H. (2007). Lost opportunities: physicians’ reasons and disparities in breast cancer treatment. J. Clin. Oncol..

[7] Cavalli-Bjorkman N. (2014). Implications of patients’ socioeconomic status - what oncologists should be aware of. Acta Oncol..

[8] Nelson H.D., Tyne K., Naik A., Bougatsos C., Chan B.K., Humphrey L. (2009). Screening for breast cancer: an update for the u.s. preventive services task force. Ann. Intern. Med..

[9] Kim J., Jang S.N. (2008). Socioeconomic disparities in breast cancer screening among US women: trends from 2000 to 2005. J. Prev. Med. Public Health.

[10] Moser K., Patnick J., Beral V. (2009). Inequalities in reported use of breast and cervical screening in Great Britain: analysis of cross sectional survey data. BMJ.

[11] Digital N.H.S. (2018). Breast Screening Programme.

[12] Peek M.E., Han J.H. (2004). Disparities in screening mammography: current status, interventions, and implications. J. Gen. Intern. Med..

[13] Zackrisson S., Lindström M., Moghaddassi M., Andersson I., Janzon L. (2007). Social predictors of non-attendance in an urban mammographic screening programme: a multilevel analysis. Scand. J. Public Health.

[14] Pruitt S.L., Shim M.J., Mullen P.D., Vernon S.W., Amick B.C. (2009). Association of area socioeconomic status and breast, cervical, and colorectal cancer screening: a systematic review. Cancer Epidemiol. Biomark. Prev..

[15] Moher D., Liberati A., Tetzlaff J., Altman D.G., Altman D., Antes G., Atkins D., Barbour V., Barrowman N. (2009). Preferred reporting items for systematic reviews and meta-analyses: the PRISMA statement. Ann. Intern. Med..

[16] Cairns J.-M., Graham E., Bambra C. (2017). Area-level socioeconomic disadvantage and suicidal behaviour in Europe: a systematic review. Soc. Sci. Med..

[17] Aarts M.J., Voogd A.C., Duijm L.E.M., Coebergh J.W.W., Louwman W.J. (2011). Socioeconomic inequalities in attending the mass screening for breast cancer in the south of the Netherlands-associations with stage at diagnosis and survival. Breast Cancer Res. Treat..

[18] Lagerlund M., Merlo J., Vicente R.P., Zackrisson S. (2015). Does the neighborhood area of residence influence non-attendance in an urban mammography screening program? A multilevel study in a Swedish city. PLoS One.

[19] Ouedraogo S., Dabakuyo-Yonli T.S., Roussot A., Pornet C., Sarlin N., Lunaud P., Desmidt P., Quantin C., Chauvin F. (2014). European transnational ecological deprivation index and participation in population-based breast cancer screening programmes in France. Prev. Med..

[20] Higgins J., Deeks J.J., Higgins J.P.T., Green S. (2011). Chapter 7: selecting studies and collecting data. Cochrane Handbook for Systematic Reviews of Interventions Version 5.1.0 [Updated March 2011].

[21] Moola S., Munn Z., Tufanaru C., Aromataris E., Sears K., Sfetcu R., Currie M., Lisy K., Qureshi R., Aromataris E., Munn Z. (2017). Chapter 7: systematic reviews of etiology and risk. Joanna Briggs Institute Reviewer’S Manual.

[22] Graham H., Kelly M. (2004). Health Inequalities: Concepts, Frameworks and Policy.

[23] Douglas E., Waller J., Duffy S.W., Wardle J. (2016). Socioeconomic inequalities in breast and cervical screening coverage in England: are we closing the gap?. J. Med. Screen..

[24] Jack R.H., Davies E.A., Robson T. (2016). The varying influence of socioeconomic deprivation on breast cancer screening uptake in London. J. Public Health.

[25] Jack R.H., Møller H., Robson T., Davies E.A. (2014). Breast cancer screening uptake among women from different ethnic groups in London: a population-based cohort study. BMJ Open.

[26] Massat N.J., Douglas E., Waller J., Wardle J., Duffy S.W. (2015). Variation in cervical and breast cancer screening coverage in England: a cross-sectional analysis to characterise districts with atypical behaviour. BMJ Open.

[27] Renshaw C., Jack R.H., Dixon S., Moller H., Davies E.A. (2010). Estimating attendance for breast cancer screening in ethnic groups in London. BMC Public Health.

[28] Deborde T., Chatignoux E., Quintin C., Beltzer N., Hamers F.F., Rogel A. (2018). Breast cancer screening programme participation and socioeconomic deprivation in France. Prev. Med..

[29] Pornet C., Dejardin O., Morlais F., Bouvier V., Launoy G. (2010). Socioeconomic and healthcare supply statistical determinants of compliance to mammography screening programs: a multilevel analysis in Calvados, France. Cancer Epidemiol..

[30] Lemke D., Berkemeyer S., Mattauch V., Heidinger O., Pebesma E., Hense H.-W. (2015). Small-area spatio-temporal analyses of participation rates in the mammography screening program in the city of Dortmund (NW Germany). BMC Public Health.

[31] Guillaume E., Launay L., Dejardin O., Bouvier V., Guittet L., Déan P., Notari A., De Mil R., Launoy G. (2017). Could mobile mammography reduce social and geographic inequalities in breast cancer screening participation?. Prev. Med..

[32] Dundar P.E., Ozyurt B.C., Erdurak K. (2012). Sociodemographic determinants of nonattendance in a population-based mammography screening program in the city of Manisa, Turkey. Sci. World J..

[33] Ozmen T., Soran A., Ozmen V. (2016). Comparison of barriers against mammography screening in socioeconomically two contrarious populations. J. Clin. Oncol..

[34] Giuliani O., Mancini S., Puliti D., Caranci N., Ravaioli A., Vattiato R., Palumbo M., Colamartini A., Biggeri A. (2016). Patterns and determinants of receipt of follow-up mammography and/or clinical examination in a cohort of Italian breast cancer survivors. Breast Cancer Res. Treat..

[35] Townsend P., Phillimore P., Beattie A. (1988). Health and Deprivation: Inequality and the North.

[36] Akinyemiju T., Ogunsina K., Sakhuja S., Ogbhodo V., Braithwaite D. (2016). Life-course socioeconomic status and breast and cervical cancer screening: analysis of the WHO’s Study on Global Ageing and Adult Health (SAGE). BMJ Open.

[37] Fukuda Y., Nakamura K., Takano T. (2005). Reduced likelihood of cancer screening among women in urban areas and with low socio-economic status: a multilevel analysis in Japan. Public Health.

[38] Robert S.A., Strombom I., Trentham-Dietz A., Hampton J.M., McElroy J.A., Newcomb P.A., Remington P.L. (2004). Socioeconomic risk factors for breast cancer: distinguishing iIndividual- and community-level effects. Epidemiology.

[39] Ferrat E., Le Breton J., Djassibel M., Veerabudun K., Brixi Z., Attali C., Renard V. (2013). Understanding barriers to organized breast cancer screening in France: women’s perceptions, attitudes, and knowledge. Fam. Pract..

[40] Anderson D., Owen T., Mairs A., McMullen C., Graham A. (2015). Barriers and motivators to participation in the Northern Ireland Breast and Cervical Screening Programmes: a qualitative study. Eur. J. Cancer Care.

[41] Australian Government Department of Health and Ageing (2008). BreastScreen Australia: Participation Qualitative Study.

[42] Collaborative Group on Hormonal Factors in Breast Cancer (1996). Breast cancer and hormonal contraceptives: collaborative reanalysis of individual data on 53 297 women with breast cancer and 100 239 women without breast cancer from 54 epidemiological studies. Lancet.

[43] Krings K.M., Matteson K.A., Allsworth J.E., Mathias E., Peipert J.F. (2008). Contraceptive choice: how do oral contraceptive users differ from condom users and women who use no contraception? Am. J. Obstet. Gynecol..

[44] Trichopoulos D., Hsieh C.C., Macmahon B., Lin T.M., Lowe C.R., Mirra A.P., Ravnihar B., Salber E.J., Salber E.J. (1983). Age at any birth and breast-cancer risk. Int. J. Cancer.

[45] Troisi R., Bjorge T., Gissler M., Grotmol T., Kitahara C.M., Saether S.M.M., Ording A.G., Skold C., Sorensen H.T. (2018). The role of pregnancy, perinatal factors and hormones in maternal cancer risk: a review of the evidence. J. Intern. Med..

[46] Phelan J.C., Link B.G., Tehranifar P. (2010). Social conditions as fundamental causes of health inequalities: theory, evidence, and policy implications. J. Health Soc. Behav..

[47] Chambers J.A., Gracie K., Millar R., Cavanagh J., Archibald D., Cook A., O’Carroll R.E. (2016). A pilot randomized controlled trial of telephone intervention to increase Breast Cancer screening uptake in socially deprived areas in Scotland (TELBRECS). J. Med. Screen..

[48] Kerrison R.S., Shukla H., Cunningham D., Oyebode O., Friedman E. (2015). Text-message reminders increase uptake of routine breast screening appointments: a randomised controlled trial in a hard-to-reach population. Br. J. Cancer.

[49] Beckfield J., Olafsdottir S., Sosnaud B. (2013). Healthcare systems in comparative perspective: classification, convergence, institutions, inequalities, and five missed turns. Annu. Rev. Sociol..

[50] Wendt C. (2014). Changing healthcare system types. Soc. Policy Adm..

[51] McPherson K. (2010). Screening for breast cancer—balancing the debate. BMJ.

