# Supplementary material for: The breast cancer paradox: A systematic review of the association between area-level deprivation and breast cancer screening uptake in Europe
Source: Cancer Epidemiol. 2019 Jun;60:77–85. doi: 10.1016/j.canep.2019.03.008 (PMC6547165; doi:10.1016/j.canep.2019.03.008)
Supplement: Supplementary file 1 [file mmc1.docx]

**Title:**

The breast cancer paradox: a systematic review of the association between area-level deprivation and breast cancer screening uptake in Europe

**Appendices:**

Appendix A: Prisma 2009 checklist

Appendix B: JBI critical appraisal checklist for analytical cross sectional studies

Appendix C: Reasons for exclusion of full‐text articles

Appendix D: JBI critical appraisal checklist scores for all included studies

Appendix A: Prisma 2009 checklist

| **Section/topic** | **#** | **Checklist item** | **Reported on page # of submitted version** |
| --- | --- | --- | --- |
| **TITLE** | | |  |
| Title | 1 | Identify the report as a systematic review, meta-analysis, or both. | 1 |
| **ABSTRACT** | | |  |
| Structured summary | 2 | Provide a structured summary including, as applicable: background; objectives; data sources; study eligibility criteria, participants, and interventions; study appraisal and synthesis methods; results; limitations; conclusions and implications of key findings; systematic review registration number. | 2 |
| **INTRODUCTION** | | |  |
| Rationale | 3 | Describe the rationale for the review in the context of what is already known. | 3-4 |
| Objectives | 4 | Provide an explicit statement of questions being addressed with reference to participants, interventions, comparisons, outcomes, and study design (PICOS). | 4 |
| **METHODS** | | |  |
| Protocol and registration | 5 | Indicate if a review protocol exists, if and where it can be accessed (e.g., Web address), and, if available, provide registration information including registration number. | 4 |
| Eligibility criteria | 6 | Specify study characteristics (e.g., PICOS, length of follow-up) and report characteristics (e.g., years considered, language, publication status) used as criteria for eligibility, giving rationale. | 4-5 |
| Information sources | 7 | Describe all information sources (e.g., databases with dates of coverage, contact with study authors to identify additional studies) in the search and date last searched. | 5 |
| Search | 8 | Present full electronic search strategy for at least one database, including any limits used, such that it could be repeated. | Table 1 |
| Study selection | 9 | State the process for selecting studies (i.e., screening, eligibility, included in systematic review, and, if applicable, included in the meta-analysis). | 5 |
| Data collection process | 10 | Describe method of data extraction from reports (e.g., piloted forms, independently, in duplicate) and any processes for obtaining and confirming data from investigators. | 5 |
| Data items | 11 | List and define all variables for which data were sought (e.g., PICOS, funding sources) and any assumptions and simplifications made. | Table 2 |
| Risk of bias in individual studies | 12 | Describe methods used for assessing risk of bias of individual studies (including specification of whether this was done at the study or outcome level), and how this information is to be used in any data synthesis. | 5-6, Appendix D |
| Summary measures | 13 | State the principal summary measures (e.g., risk ratio, difference in means). | 4-5 |
| Synthesis of results | 14 | Describe the methods of handling data and combining results of studies, if done, including measures of consistency (e.g., I^2^) for each meta-analysis. | NA |
| Risk of bias across studies | 15 | Specify any assessment of risk of bias that may affect the cumulative evidence (e.g., publication bias, selective reporting within studies). | 17 |
| Additional analyses | 16 | Describe methods of additional analyses (e.g., sensitivity or subgroup analyses, meta-regression), if done, indicating which were pre-specified. | NA |
| **RESULTS** | | |  |
| Study selection | 17 | Give numbers of studies screened, assessed for eligibility, and included in the review, with reasons for exclusions at each stage, ideally with a flow diagram. | Figure 1 |
| Study characteristics | 18 | For each study, present characteristics for which data were extracted (e.g., study size, PICOS, follow-up period) and provide the citations. | Table 2 |
| Risk of bias within studies | 19 | Present data on risk of bias of each study and, if available, any outcome level assessment (see item 12). | Appendix D |
| Results of individual studies | 20 | For all outcomes considered (benefits or harms), present, for each study: (a) simple summary data for each intervention group (b) effect estimates and confidence intervals, ideally with a forest plot. | Table 2 |
| Synthesis of results | 21 | Present results of each meta-analysis done, including confidence intervals and measures of consistency. | NA |
| Risk of bias across studies | 22 | Present results of any assessment of risk of bias across studies (see Item 15). | NA |
| Additional analysis | 23 | Give results of additional analyses, if done (e.g., sensitivity or subgroup analyses, meta-regression [see Item 16]). | NA |
| **DISCUSSION** | | |  |
| Summary of evidence | 24 | Summarize the main findings including the strength of evidence for each main outcome; consider their relevance to key groups (e.g., healthcare providers, users, and policy makers). | 9-11 |
| Limitations | 25 | Discuss limitations at study and outcome level (e.g., risk of bias), and at review-level (e.g., incomplete retrieval of identified research, reporting bias). | 9-11 |
| Conclusions | 26 | Provide a general interpretation of the results in the context of other evidence, and implications for future research. | 9-11 |
| **FUNDING** | | |  |
| Funding | 27 | Describe sources of funding for the systematic review and other support (e.g., supply of data); role of funders for the systematic review. | 18 |

*From:*  Moher D, Liberati A, Tetzlaff J, Altman DG, The PRISMA Group (2009). Preferred Reporting Items for Systematic Reviews and Meta-Analyses: The PRISMA Statement. PLoS Med 6(6): e1000097. doi:10.1371/journal.pmed1000097

For more information, visit: **www.prisma-statement.org**.

Appendix B:

**JBI Critical Appraisal Checklist for Analytical Cross Sectional Studies**

Reviewer: ………………………………………………………… Date: ………………………………………………

Author: ……………………………………………………………. Year: ………………………………………………

|  | Yes | No | Unclear | Not applicable |
| --- | --- | --- | --- | --- |
| 1. Were the criteria for inclusion in the sample clearly defined? |  |  |  |  |
| 2. Were the study subjects and the setting described in detail? |  |  |  |  |
| 3. Was the exposure measured in a valid and reliable way? |  |  |  |  |
| 4. Were objective, standard criteria used for measurement of the condition |  |  |  |  |
| 5. Were confounding factors identified? |  |  |  |  |
| 6. Were strategies to deal with confounding factors stated? |  |  |  |  |
| 7. Were the outcomes measured in a valid and reliable way? |  |  |  |  |
| 8. Was appropriate statistical analysis used? |  |  |  |  |

Overall appraisal: Include Exclude Seek further info

Comments (including reason for exclusion): ……………………………………………………………………………………………………………………………………………………………………………………………………………………..…………………………………………………………………………………………………………………………………………………………………………………………………………………………………………………………………..…………………………………………………………………

Appendix C: Reasons for exclusion of full‐text articles

Detailed below are full references for all excluded articles. Reasons for exclusion are given at the start of each section. Articles are excluded for one of the following reasons (in order of hierarchical importance as set out below):

1. Incorrect population
2. No relevant intervention
3. No area-level deprivation provided
4. No relevant outcome data
5. Insufficient information provided
6. Study not in English

**Incorrect population**

Bouton ME, Nodora J, Hsu C-H, Green A, Martinez ME, Komenaka IK: **Understanding of breast cancer concepts in an undereducated county hospital population**. *Journal of Surgical Oncology* 2010, **102**(5):398-403.

Davey-Rothwell MA, Bowie J, Murray L, Latkin CA: **Perceptions of one's neighborhood and mammogram use among a sample of low-income women at risk for human immunodeficiency virus and sexually transmitted infections**. *Women's Health Issues* 2016, **26**(2):196-200.

de Oliveira, RDP, Santos MCL, Moreira CB, Fernandes AFC: **Detection of breast cancer: knowledge, attitude, and practice of family health strategy women**. *Journal of Cancer Education* 2018, **33**(5): 1082-1087.

Harper S, Lynch J, Meersman SC, Breen N, Davis WW, Reichman MC: **Trends in area-socioeconomic and race-ethnic disparities in breast cancer incidence, stage at diagnosis, screening, mortality, and survival among women ages 50 years and over (1987-2005)**. *Cancer Epidemiology, Biomarkers & Prevention* 2009, **18**(1):121-131.

Khan N, Kaestner R, Salmon JW, Gutierrez B: **Does supply influence mammography screening?** *American journal of health behavior* 2010, **34**(4):465-475.

Lian M, Struthers J, Schootman M: **Comparing GIS-based measures in access to mammography and their validity in predicting neighborhood risk of late-stage breast cancer**. *PloS One* 2012, **7**(8):e43000.

Moss JL, Liu, B, Feuer EJ: **Urban/rural differences in breast and cervical cancer incidence: the mediating roles of socioeconomic status and provider density**. *Women's Health Issues* 2017, **27**(6): 683-691.

Rahman SMM, Rahman S, Dignan MB, Lindquist PS: **Access to mammography facilities and breast cancer stage at diagnosis: does geographic distance predict?** *International Journal of Cancer Prevention* 2010, **3**(3):137-147.

Rakowski W, Clark MA, Rogers ML, Weitzen SH: **Reversals of association for pap, colorectal, and prostate cancer testing among Hispanic and non-Hispanic Black women and men**. *Cancer Epidemiology Biomarkers and Prevention* 2011, **20**(5):876-889.

Williams BA, Lindquist K, Sudore RL, Covinsky KE, Walter LC: **Screening mammography in older women: effect of wealth and prognosis**. *Archives of Internal Medicine* 2008, **168**(5):514-520.

**No relevant intervention**

Abali H, Ata A, Gokce G, Gokce H: **Possible logistic and sociodemographic factors on breast cancer screening in Turkey: lessons from a women's health project in Mersin province**. *Journal of Cancer Education* 2012, **27**(2):347-352.

Morris M, Woods LM, Bhaskaran K, Rachet B: **Do pre-diagnosis primary care consultation patterns explain deprivation-specific differences in net survival among women with breast cancer? An examination of individually-linked data from the UK West Midlands cancer registry, national screening programme and Clinical Practice Research Datalink**. *BMC Cancer* 2017, **17**(1):155.

Viel J-F, Rymzhanova R: **Mammographic density and urbanization: a population-based screening study**. *Journal of Medical Screening* 2012, **19**(1):20-25.

Zengarini N, Ponti A, Tomatis M, Casella D, Giordano L, Mano MP, Segnan N, Whitehead M, Costa G, Spadea T: **Absence of socioeconomic inequalities in access to good-quality breast cancer treatment within a population-wide screening programme in Turin (Italy)**. *European Journal of Cancer Prevention* 2016, **25**(6):538-546.

**No area-level deprivation provided**

Bansal N, Bhopal RS, Steiner MFC, Brewster DH: **Major ethnic group differences in breast cancer screening uptake in Scotland are not extinguished by adjustment for indices of geographical residence, area deprivation, long-term illness and education**. *British Journal of Cancer* 2012, **106**(8):1361-1366.

Brumback BA, Cai Z, Dailey AB: **Methods of estimating or accounting for neighborhood associations with health using complex survey data**. *American Journal of Epidemiology* 2014, **179**(10):1255-1263.

Carrieri V, Wuebker A: **Assessing inequalities in preventive care use in Europe**. *Health Policy* 2013, **113**(3):247-257.

Carrozzi G, Sampaolo L, Bolognesi L, Sardonini L, Bertozzi N, Giorgi Rossi P, Zappa M, Baldissera S, Campostrini S, Ferrante G *et al*: **Cancer screening uptake: association with individual characteristics, geographic distribution, and time trends in Italy**. *Epidemiologia e Prevenzione* 2015, **39**(3 Suppl 1):9-18.

Chambers JA, Gracie K, Millar R, Cavanagh J, Archibald D, Cook A, O’Carroll RE: **A pilot randomized controlled trial of telephone intervention to increase breast cancer screening uptake in socially deprived areas in Scotland (TELBRECS).** *Journal of Medical Screening* 2016, **23**(3): 141-149.

Chkotua S, Peleteiro B: **Mammography use in Portugal: National Health Survey 2014**. *Preventing Chronic Disease* 2017, **14**: E100.

Coyle C, Kinnear H, Rosato M, Mairs A, Hall C, O'Reilly D: **Do women who intermittently attend breast screening differ from those who attend every invitation and those who never attend?** *Journal of Medical Screening* 2014, **21**(2):98-103.

Cullati S, von Arx M, Courvoisier DS, Sandoval JL, Manor O, Burton-Jeangros C, Bouchardy C, Guessous I: **Organised population-based programmes and change in socioeconomic inequalities in mammography screening: a 1992-2012 nationwide quasi-experimental study**. *Preventive Medicine* 2018, 116: 19-26.

Deandrea S, Molina-Barcelo A, Uluturk A, Moreno J, Neamtiu L, Peiro-Perez R, Saz-Parkinson Z, Lopez-Alcalde J, Lerda D, Salas D: **Presence, characteristics and equity of access to breast cancer screening programmes in 27 European countries in 2010 and 2014. Results from an international survey**. *Preventive Medicine* 2016, **91**: 250-263.

De la Cruz-Sanchez E, Aguirre-Gomez L: **Health related lifestyle and preventive medical care of rural Spanish women compared to their urban counterparts**. *Journal of Immigrant and Minority Health* 2014, **16**(4):712-718.

Dourado F, Carreira H, Lunet N: **Mammography use for breast cancer screening in Portugal: results from the 2005/2006 National Health Survey**. *European Journal of Public Health* 2013, **23**(3):386-392.

Duport N, Ancelle-Park R, Boussac-Zarebska M, Uhry Z, Bloch J: **Are breast cancer screening practices associated with sociodemographic status and healthcare access? Analysis of a French cross-sectional study**. *European Journal of Cancer Prevention* 2008, **17**(3):218-224.

Fenner L, Kassner A, Berlin C, Egger M, Zwahlen M: **Trends in the use of mammography for early breast cancer detection in Switzerland: Swiss Health Surveys 2007 and 2012**. *Swiss Medical Weekly* 2018, 148: w14603.

Garrido-Cumbrera M, Borrell C, Palencia L, Espelt A, Rodriguez-Sanz M, Pasarin MI, Kunst A: **Social class inequalities in the utilization of health care and preventive services in Spain, a country with a national health system**. *International Journal of Health Services* 2010, **40**(3):525-542.

Gobl CS, Ortag F, Bozkurt L, Smeikal A, Dadak C, Kautzky-Willer A: **Health behaviour and attitude towards screening examinations in an Austrian urban and rural population: gender aspects - screening and sex**. *Wiener Medizinische Wochenschrift* 2011, **161**(5-6):143-148.

Guillaume E, Launay L, Dejardin O, Bouvier V, Guittet L, Dean P, Notari A, De Mil R, Launoy G: **Could mobile mammography reduce social and geographic inequalities in breast cancer screening participation?** *Preventive Medicine* 2017, **100**:84-88.

Hellquist BN, Czene K, Hjalm A, Nystrom L, Jonsson H: **Effectiveness of population-based service screening with mammography for women ages 40 to 49 years with a high or low risk of breast cancer: socioeconomic status, parity, and age at birth of first child**. *Cancer* 2015, **121**(2):251-258.

Jensen LF, Pedersen AF, Andersen B, Vedsted P: **Identifying specific non-attending groups in breast cancer screening--population-based registry study of participation and socio-demography**. *BMC Cancer* 2012, **12**:518.

Kinnear H, Rosato M, Mairs A, Hall C, O'Reilly D: **The low uptake of breast screening in cities is a major public health issue and may be due to organisational factors: a Census-based record linkage study**. *The Breast* 2011, **20**(5):460-463.

Kristiansen M, Thorsted BL, Krasnik A, von Euler-Chelpin M: **Participation in mammography screening among migrants and non-migrants in Denmark**. *Acta Oncologica* 2012, **51**(1):28-36.

Leung J, Macleoad C, McLaughlin D, Woods L, Henderson R, Watson A, Kyle R, Hubbard G, Mullen R, Atherton I: **Rural-urban differences in screening mammography uptake in Australia and Scotland**. *European Journal of Surgical Oncology* 2015, **41**(6):S28.

Lopez-De-Andres A, Martin-Lopez R, Hernandez-Barrera V, Carrasco-Garrido P, Gil-De-Miguel A, Esteban YPMM, Jimenez-Garcia R: **Predictors of breast and cervical cancer screening in a Spanish metropolitan area**. *Journal of Women's Health* 2010, **19**(9):1675-1681.

Mandelzweig L, Chetrit A, Amitai T, Silverman B, Siegelmann-Danieli N, Sadetzki S: **Primary prevention and screening practices among long-term breast cancer survivors**. *Cancer Causes & Control* 2017, **28**(7): 657-666.

Menvielle G, Richard J-B, Ringa V, Dray-Spira R, Beck F: **To what extent is women's economic situation associated with cancer screening uptake when nationwide screening exists? A study of breast and cervical cancer screening in France in 2010**. *Cancer Causes & Control* 2014, **25**(8):977-983.

Moser K, Patnick J, Beral V: **Inequalities in reported use of breast and cervical screening in Great Britain: analysis of cross sectional survey data**. *BMJ* 2009, **338**:b2025.

Orsini M, Tretarre B, Daures JP, Bessaoud F: **Individual socioeconomic status and breast cancer diagnostic stages: a French case-control study**. *European Journal of Public Health* 2016, 26(3): 445-450.

Padoan M, Ferrante D, Pretti G, Magnani C: **Study of socio-economic characteristics, diagnosis and outcome of women participating or not participating in mammogram screening**. *Annali di Igiene* 2014, **26**(6):518-526.

Palencia L, Espelt A, Rodriguez-Sanz M, Puigpinos R, Pons-Vigues M, Pasarin MI, Spadea T, Kunst AE, Borrell C: **Socio-economic inequalities in breast and cervical cancer screening practices in Europe: influence of the type of screening program**. *International Journal of Epidemiology* 2010, **39**(3):757-765.

Petrelli A, Giorgi Rossi P, Francovich L, Giordani B, Di Napoli A, Zappa M, Mirisola C, Gargiulo L: **Geographical and socioeconomic differences in uptake of Pap test and mammography in Italy: results from the National Health Interview Survey**. BMJ Open 2018, **8**(9): e021653.

Pons-Vigues M, Puigpinos-Riera R, Rodriguez-Sanz M, Serral G, Palencia L, Borrell C: **Preventive control of breast and cervical cancer in immigrant and native women in Spain: the role of country of origin and social class**. *International Journal of Health Services* 2011, **41**(3):483-499.

Relecom A, Arzel B, Perneger T: **Effect of an organised screening program on socioeconomic inequalities in mammography practice, knowledge and attitudes**. *International Journal for Equity in Health* 2018, **17**(1): 95.

Ricardo-Rodrigues I, Jimenez-Garcia R, Hernandez-Barrera V, Carrasco-Garrido P, Jimenez-Trujillo I, Lopez de Andres A: **Social disparities in access to breast and cervical cancer screening by women living in Spain**. *Public Health* 2015, **129**(7):881-888.

Rondet C, Lapostolle A, Soler M, Grillo F, Parizot I, Chauvin P: **Are immigrants and nationals born to immigrants at higher risk for delayed or no lifetime breast and cervical cancer screening? The results from a population-based survey in Paris metropolitan area in 2010**. *PloS One* 2014, **9**(1):e87046.

Sandoval JL, Himsl R, Theler JM, Gaspoz JM, Joost S, Guessous I: **Spatial distribution of mammography adherence in a Swiss urban population and its association with socioeconomic status**. *Cancer Medicine* 2018, **7**(12): 6299-6307.

Sozmen K, Unal B, Sakarya S, Dinc G, Yardim N, Keskinkilic B, Ergor G: **Determinants of breast and cervical cancer screening uptake among women in Turkey**. *Asia-Pacific Journal of Public Health* 2016, **28**(6):528-538.

Stamenic V, Strnad M: **Urban-rural differences in a population-based breast cancer screening program in Croatia**. *Croatian Medical Journal* 2011, **52**(1):76-86.

Ucuncu MZ, Ucuncu MM, Toprak D: Evaluation knowledge, attitude, and behaviour for breast cancer among young women living in two different habitats of Turkey. **Asian Pacific Journal of Cancer Prevention** 2018, **19**(11): 3179-3185.

Vogt V, Siegel M, Sundmacher L: **Examining regional variation in the use of cancer screening in Germany**. *Social Science and Medicine* 2014, **110**:74-80.

Walsh B, Silles M, O'Neill C: **The importance of socio-economic variables in cancer screening participation: a comparison between population-based and opportunistic screening in the EU-15**. *Health Policy* 2011, **101**(3):269-276.

Willems B, Bracke P: **The impact of regional screening policies on the diffusion of cancer screening participation in Belgium: time trends in educational inequalities in Flanders and Wallonia**. *BMC Health Services Research* 2018, **18**(1): 943.

Willems B, Bracke P: Participants, physicians or programmes: participants' educational level and initiative in cancer screening. *Health Policy* 2018, **122**(4): 422-430.

Zidar MN, Larm P, Tillgren P, Akhavan S: **Non-attendance of mammographic screening: the roles of age and municipality in a population-based Swedish sample**. *International Journal for Equity in Health* 2015, **14**:157.

**No relevant outcome data**

Cuthbertson SA, Goyder EC, Poole J: **Inequalities in breast cancer stage at diagnosis in the Trent region, and implications for the NHS Breast Screening Programme**. *Journal of Public Health* 2009, **31**(3):398-405.

Davies EA, Renshaw C, Dixon S, Moller H, Coupland VH: **Socioeconomic and ethnic inequalities in screen-detected breast cancer in London**. *Journal of Public Health* 2013, **35**(4):607-615.

Herrmann C, Vounatsou P, Thurlimann B, Probst-Hensch N, Rothermundt C, Ess S: **Impact of mammography screening programmes on breast cancer mortality in Switzerland, a country with different regional screening policies**. BMJ Open 2018, **8**(3): e017806.

Morris M, Woods LM, Rachet B: **What might explain deprivation-specific differences in the excess hazard of breast cancer death amongst screen-detected women? Analysis of patients diagnosed in the West Midlands region of England from 1989 to 2011**. *Oncotarget* 2016, **7**(31):49939-49947.

Schootman M, Lian M, Deshpande AD, Baker EA, Pruitt SL, Aft R, Jeffe DB: **Temporal trends in area socioeconomic disparities in breast-cancer incidence and mortality, 1988-2005**. *Breast Cancer Research and Treatment* 2010, **122**(2):533-543.

Simou E, Foundoulakis E, Kourlaba G, Maniadakis N: **Factors associated with the use of preventive services by women in Greece**. *European Journal of Public Health* 2011, **21**(4):512-519.

Woods LM, Morris M, Rachet B: **No 'cure' within 12 years of diagnosis among breast cancer patients who are diagnosed via mammographic screening: women diagnosed in the West Midlands region of England 1989-2011**. *Annals of Oncology* 2016, **27**(11): 2025-2031.

**Insufficient data**

Gomez K, Micic T, Barnes R: **Inequalities in health: not the case for breast cancer in South-East Wales?** *European Journal of Cancer* 2012, **48**:S76.

Hassiotis A, Osborn D, Horsfall L, Petersen I, Walters K, Nazareth I: **Access to cancer screening in people with learning disabilities in the UK using a primary care database**. *Journal of Intellectual Disability Research* 2012, **56**(7-8):763.

Huang DT, Hines R: **Disparities in cancer screening and death rates**. *American Journal of Epidemiology* 2012, **175**:S102.

Immonen-Raiha P, Kauhava L, Heinavaara S, Anttila A, Pylkkanen L, Klemi P, Parvinen I: **Long-term follow-up of breast cancer mortality in Turku, Helsinki and the rest of Finland with employment of different screening policies**. *Asia-Pacific Journal of Clinical Oncology* 2014, **10**:11.

Jelastopulu E, Lempotesi K, Bartsokas C, Karnaki P, Sissouras A: **Factors associated with mammography screening in Patras, Western Greece**. *European Journal of Epidemiology* 2012, **27**(1 SUPPL. 1):S158.

Khan H, Meraj S, Wilbraham A, Cox D, Bhatt R, Yates J, Waldron J, Powell A: **PB.43: Review of the determinants of poor screening uptake at City, Sandwell and Walsall Breast Screening Units and the steps taken to improve attendance**. *Breast Cancer Research* 2013, **15**(Supplement 1):P43.

Ozmen V: **Screening mammography in Turkey and developed countries: controversial issues**. *Psycho-Oncology* 2011, **20**(S2):80-81.

Puigpinos-Riera R, Serral G, Borrell C, Pons-Vigues M, Macia F, Puig MT, Bargallo X, Martinez X: **The programme of breast cancer screening in Barcelona: begin and evolution. Where are we now?** *European Journal of Cancer, Supplement* 2010, **8**(3):244.

**Study not in English**

Albert US, Kalder M, Schulte H, Klusendick M, Diener J, Schulz-Zehden B, Kopp I, Nass-Griegoleit I: **The population-based mammography screening programme in Germany: uptake and first experiences of women in 10 federal states [In German]**. *Gesundheitswesen* 2012, **74**(2):61-70.

Burrion JB: **[Breast cancer screening: present situation and prospects]**." *Revue Medicale de Bruxelles* 2018, **39**(4): 406-409.

Jokiel M: **Social aspects of breast cancer early detection after introduction of population screenings in Poland [In Polish]**. *Spoleczne aspekty wczesnego wykrywania raka piersi po wprowadzeniu w Polsce skryningow populacyjnych* 2009, **63**(3):443-447.

Ouedraogo S, Dabakuyo-Yonli TS, Roussot A, Dialla PO, Pornet C, Poillot ML, Soler-Michel P, Sarlin N, Lunaud P, Desmidt P *et al*: **Breast cancer screening in thirteen French departments [In French]**. *Bulletin du Cancer* 2015, **102**(2):126-138.

Serral G, Borrell C, Puigpinos I, Riera RG: **[Socioeconomic inequalities in mammography screening in Spanish women aged 45 to 69].** *Gaceta Sanitaria* 2018, **32**(1): 61-67.

Tornberg S, Lidbrink E, Henriksson R: **Free of charge mammography gets more people to the examination. Study in Stockholm County shows good efficacy in socioeconomically deprivation areas [In Swedish]**. *Avgiftsfri mammografi far fler att komma till undersokning Studie i Stockholms lan visar pa bra effekt i socioekonomiskt svaga omraden* 2014, **111**(7):278-281.

Appendix D: JBI critical appraisal checklist scores for all included studies.

| **JBI Critical Appraisal Checklist** | Massat et al. (2015) | Renshaw et al. (2010) | Jack et al. (2014, 2016)^a^ | Douglas et al. (2016) | Pornet et al. (2010) | Ouedraogo et al. (2014) | Deborde et al. (2018) | Lemke et al. (2015) | Giuliani et al. (2016) | Aarts et al. (2011) | Lagerland et al. (2015) | Dundar et al. (2015) | Ozmen et al. (2016) |
| --- | --- | --- | --- | --- | --- | --- | --- | --- | --- | --- | --- | --- | --- |
| Were the criteria for inclusion in the sample clearly defined? |  |  |  |  |  |  |  |  |  |  |  |  |  |
| Were the study subjects and the setting described in detail? |  |  |  |  |  |  |  |  |  |  |  |  |  |
| Was the exposure measured in a valid and reliable way? |  |  |  |  |  |  |  |  |  |  |  |  |  |
| Were objective, standard criteria used for measurement of the condition? |  |  |  |  |  |  |  |  |  |  |  |  |  |
| Were confounding factors identified? |  |  |  |  |  |  |  |  |  |  |  |  |  |
| Were strategies to deal with confounding factors stated? |  |  |  |  |  |  |  |  |  |  |  |  |  |
| Were the outcomes measured in a valid and reliable way? |  |  |  |  |  |  |  |  |  |  |  |  |  |
| Was appropriate statistical analysis used? |  |  |  |  |  |  |  |  |  |  |  |  |  |
| **SUMMARY** | High | Medium | Medium | Medium | High | High | Medium | High | High | Medium | High | Low | Low |

^a^ Aggregate scores for both articles representing single study.
